# Advancements in aptasensors development for the detection of zearalenone

**DOI:** 10.3389/fnut.2026.1778772

**Published:** 2026-03-09

**Authors:** Sihui Cheng, Junjie Xu, Pengfei Gao, Jingjing Cai, Yang Cao, Yiwei Hong, Hao Ding, Hao Chen, Xu Liu, Genxi Zhang, Tao Zhang, Xiaodong Guo, Kaizhou Xie

**Affiliations:** 1 College of Animal Science and Technology, Yangzhou University, Yangzhou, Jiangsu, China; 2 Joint International Research Laboratory of Agriculture and Agri-Product Safety, Yangzhou University, Yangzhou, Jiangsu, China; 3 College of Veterinary Medicine, Yangzhou University, Yangzhou, Jiangsu, China

**Keywords:** aptamer, aptasensor, detection, food safety, zearalenone

## Abstract

Zearalenone (ZEN), an estrogenic mycotoxin produced by *Fusarium* species, is a frequent contaminant in cereals and other agricultural commodities. Its presence poses substantial economic losses, threatens food safety, and presents significant risks to human health, making it a growing concern worldwide. Consequently, the development of rapid, sensitive, and reliable methods for ZEN detection is of critical importance. In recent years, aptasensors technologies have emerged as promising tools for mycotoxin monitoring. This review comprehensively summarizes recent advances in ZEN-specific aptasensors, with a focus on detection mechanisms, analytical performance, and comparative advantages and limitations of various platforms. By providing an up-to-date overview of optical, electrochemical, and other emerging sensing strategies, this work aims to support future developments in mycotoxin prevention and detection, thereby contributing to enhanced food safety assurance.

## Graphical abstract

**Figure:**
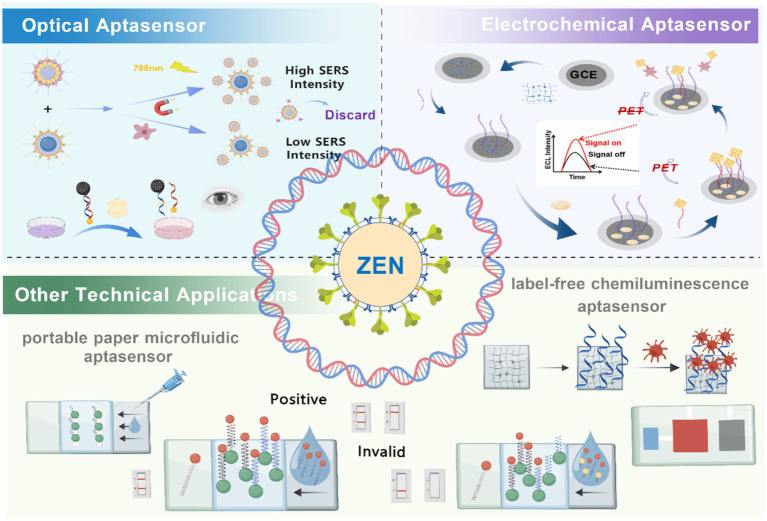

## 1 Introduction

Zearalenone is a mycotoxin produced by the biosynthesis of various fungi and frequently appears in our environment as a contaminant (1). It exhibits potent estrogenic effects in animals by binding to specific estrogen receptors, disrupting reproductive hormone levels and causing a range of reproductive disorders (2–6). These effects are commonly observed in livestock, manifesting as reduced fertility, smaller litter sizes, and abnormal fetal development (7, 8). Moreover, ZEN metabolites often exhibit even greater toxicity (9), with adverse impacts increasingly reported at cellular, immune, hepatic, renal, and genetic levels (10–15). Human exposure to ZEN occurs not only through contaminated grains but also via various derived products, such as alcoholic beverages made from contaminated cereals, milk from dairy cows fed ZEN-contaminated feed, and meat products from affected livestock (16, 17). Due to its health risks, ZEN has been classified as a Group III carcinogen by the International Agency for Research on Cancer (IARC), and many countries have established regulatory limits for its presence in food and feed (18, 19). In recent years, environmental changes have contributed to a marked increase in the contamination rates of food and crops, underscoring the urgent need for effective monitoring to prevent further significant losses (20). The inherent stability of ZEN, along with its insolubility in water and resistance to high temperatures, makes it persistent and difficult to degrade during conventional feed and food processing (21, 22).

**Figure 1:**
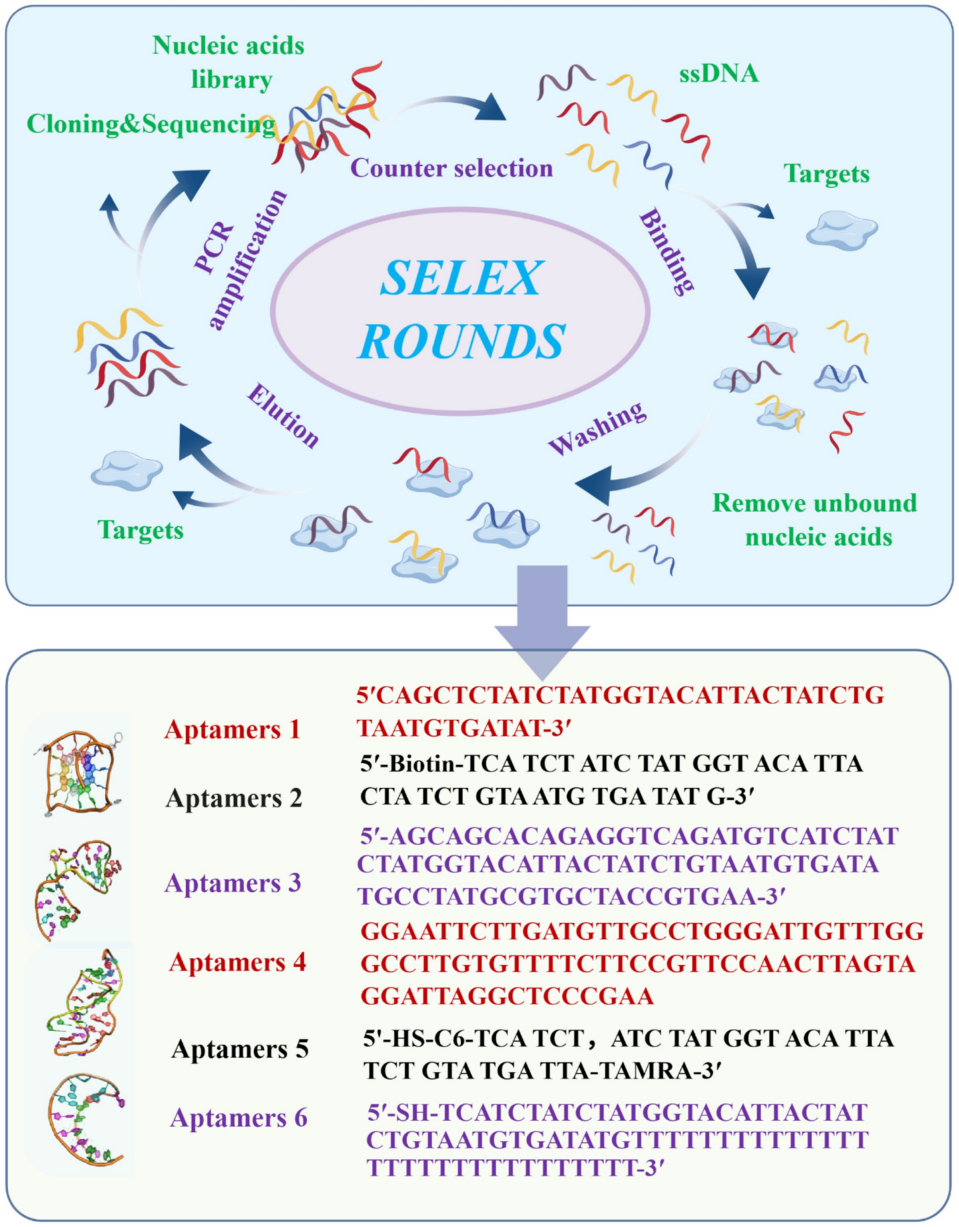
Aptamer screening based on SELEX technology and common ZEN Aptamer sequences (68, 73, 115, 124–126).

Recent advances in detection methodologies have introduced novel strategies that enhance molecular recognition, reduce assay time, and improve signal amplification (23). Among these, aptasensors have gained significant prominence. Aptamers are short, single-stranded oligonucleotides that can bind with high specificity and affinity to diverse targets, ranging from small molecules to complex proteins (24–27). They offer notable advantages, including high stability, cost-effectiveness, and long shelf life (28). Furthermore, ongoing technological innovations are making aptasensors increasingly portable, user-friendly, and affordable, enabling their application even by non-specialists (29). Currently, aptasensors are widely applied in mycotoxin detection and are primarily categorized into optical, electrochemical, and other emerging biosensing platforms. Optical aptasensors exploit the light absorption or emission properties of target molecules, allowing highly sensitive and rapid detection. Electrochemical aptasensors monitor current changes resulting from reactions at electrode surfaces, offering benefits such as low cost and fast response. Other aptasensors, including label-free chemiluminescent sensors, optical fiber-based sensors, and multiplex platforms capable of simultaneous detection of multiple toxins, show considerable potential for real-world applications in agriculture, food safety, and environmental monitoring. Among these, aptamer-based ZEN detectors are frequently employed (Figure 1).

In recent years, the integration of advanced materials and biomolecules, such as nanomaterials and enzymes, has significantly enhanced the performance of aptasensors. Nanomaterials improve sensor reactivity and surface characteristics, while enzymes amplify signals through catalytic reactions, collectively boosting sensitivity, selectivity, and response speed. These hybrid systems enable more efficient, accurate, and rapid detection of target molecules. This review summarizes the principles, recent developments, and future directions of novel aptasensors for ZEN detection, aiming to support further advancements in food safety testing.

## 2 Signal transduction strategies

### 2.1 Optical signal transduction strategy

**Figure 2:**
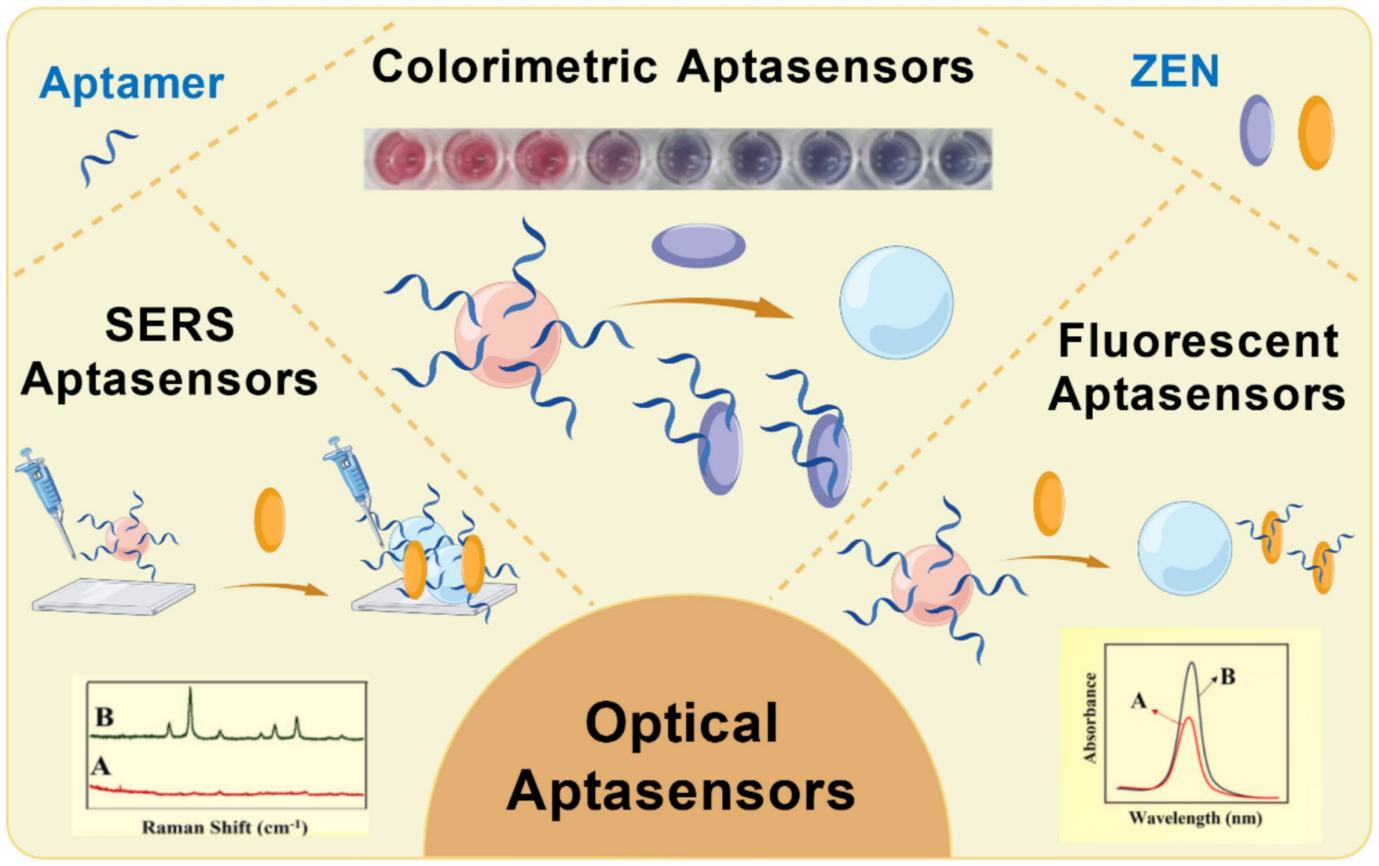
The types of optical aptasensors for detection of ZEN.

Optical signal transduction is a widely used method in aptasensors, which monitors the binding event between an aptamer and its target molecule by exploiting various optical properties. As illustrated in Figure 2, common optical approaches include fluorescence, colorimetry, and surface-enhanced Raman scattering (SERS).

In fluorescence-based aptasensors, signal transduction typically relies on changes in fluorescence emission resulting from the aptamer-target interaction. This is often achieved by labeling the aptamer with a fluorescent probe. Upon target binding, a change in fluorescence signal, either in intensity or emission wavelength, occurs, which can be quantitatively measured to determine target concentration (30). This method offers high sensitivity and rapid response, making it suitable for applications such as biomedical analysis and mycotoxin detection.

Colorimetric sensors transduce signals through visible color changes induced by the binding of the target molecule to the aptamer. These systems frequently employ aptamers functionalized with nanomaterials such as gold nanoparticles or carbon nanotubes. The aggregation or dispersion of these nanomaterials upon target binding leads to a distinct color shift, enabling the quantification of the target molecule by measuring the extent of color variation (31). Colorimetric methods are valued for their operational simplicity and low cost, making them ideal for on-site rapid screening.

Another prominent technique is SERS. SERS is a powerful spectroscopic tool that significantly enhances Raman scattering signals through electromagnetic enhancement from nanostructured substrates. Recent advances have focused on the development of novel nanostructures, including gold (AuNPs) and silver nanoparticles (AgNPs), to maximize this enhancement effect (32). By combining the molecular specificity of aptamers with the signal amplification provided by SERS, this approach allows for the highly sensitive detection of target molecules even at trace levels. As a result, SERS-based aptasensors are increasingly applied across biological, chemical, and environmental monitoring fields.

### 2.2 Electrochemical signal transduction strategy

**Figure 3:**
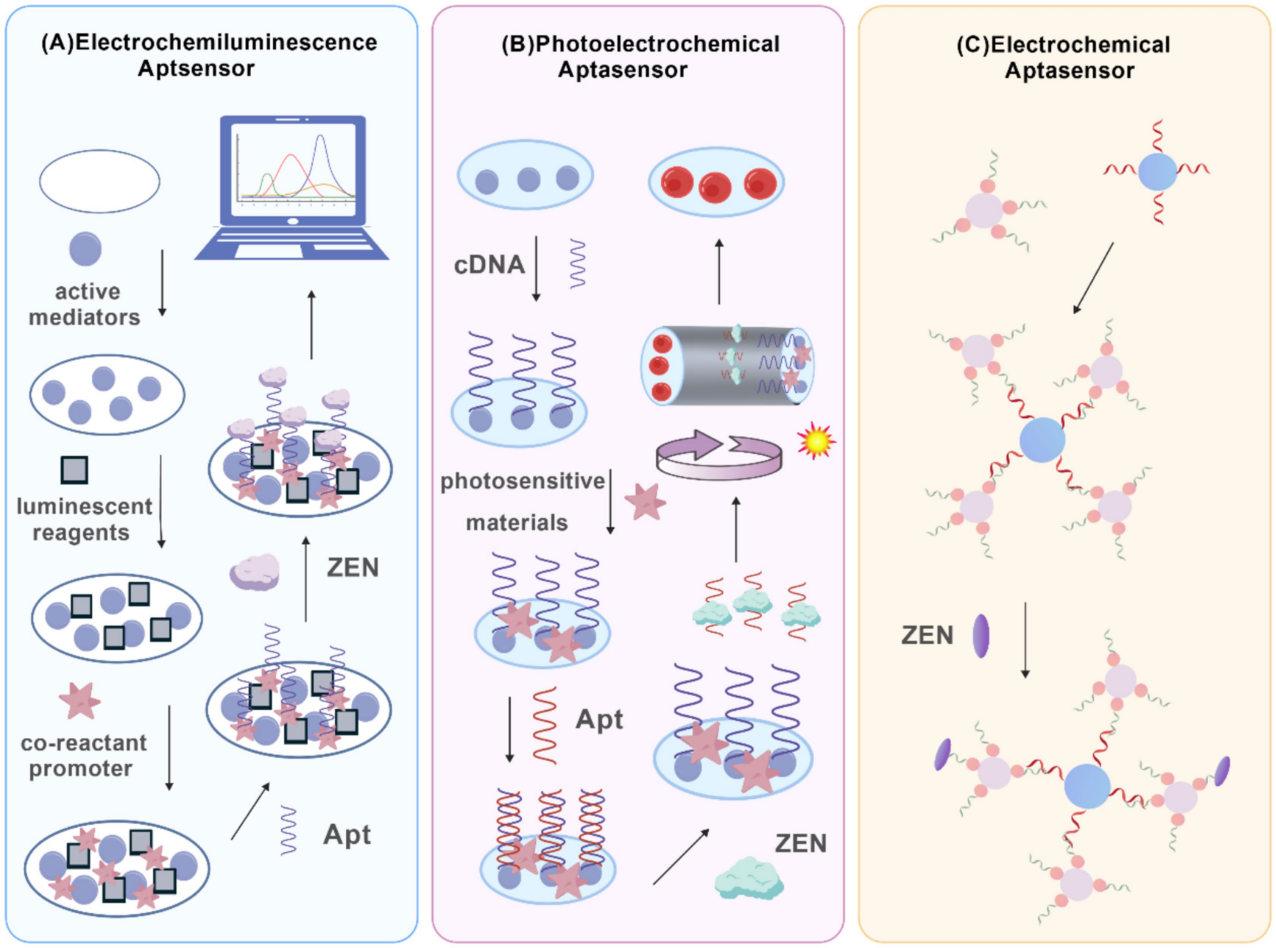
The types of electrochemical aptasensors, including **(A)** ECl aptasensor, **(B)** PEC aptasensor, and **(C)** EC aptasensor.

Electrochemical signal transduction serves as a fundamental and highly adaptable strategy in aptasensor design. This approach detects the binding between an aptamer and its target by measuring corresponding changes in electrical parameters such as current, potential, or impedance, which result from binding-induced modifications at the electrode interface (33). Typical implementations of electrochemical aptasensors for ZEN detection are presented in Figure 3, including electrochemiluminescence (ECL), photoelectrochemical (PEC), and direct electrochemical (EC) aptasensors. Owing to their intrinsic advantages of rapid response, high sensitivity, and ease of miniaturization, electrochemical aptasensors have been extensively applied in the field of mycotoxin analysis (34).

The operational principles of these sensors are realized through distinct electrochemical techniques. Voltammetric techniques rely on binding-induced modifications in electron transfer kinetics at the electrode surface. The specific conformational change of the aptamer upon target binding can either facilitate or hinder electron transfer, resulting in a quantifiable change in the Faradaic current. This current signal, which is proportional to the target concentration, provides a highly sensitive basis for detection, making voltammetric aptasensors particularly suitable for portable and real-time monitoring (35). In electrochemical impedance spectroscopy (EIS), the binding event is transduced into an increase in electron transfer resistance. The formation of the aptamer-target complex alters the interfacial characteristics of the electrode, impeding the flow of electrons. By analyzing the resulting impedance shift using established current–voltage relationships, EIS offers a powerful, label-free detection mechanism. EIS-based aptasensors are widely valued for their robust stability and excellent reproducibility (36, 37). Furthermore, potentiometric aptasensors detect binding through shifts in the equilibrium potential at the electrode-solution interface. The association of the aptamer with its target disrupts the local charge distribution, generating a measurable potential change that correlates directly with analyte concentration. This mode provides high specificity and is considered highly suitable for precise toxin risk assessment and other demanding analytical applications (38).

### 2.3 Magnetic signal transduction strategy

The magnetic signal transduction strategy represents an emerging approach in aptasensor design, utilizing the unique properties of magnetic materials for detection. A common implementation involves labeling aptamers with magnetic nanoparticles (MNPs), such as Fe_3_O_4_ particles (39). When the aptamer binds to its target, the resulting complex alters the distribution or quantity of MNPs in the sensing system. These changes can be precisely measured under an external magnetic field, enabling quantitative detection of the target analyte. A key advantage of magnetic transduction is its strong anti-interference capability against matrix effects, making it particularly suitable for analyzing complex biological and environmental samples.

### 2.4 Composite sensor transduction strategy

With the continuous development of sensor technology, the limitations of relying on a single transduction mechanism have become increasingly apparent. To address this, composite sensing strategies have been developed, integrating multiple signal transduction modalities such as optical, electrochemical, and magnetic methods into a single platform. This multi-dimensional detection approach provides more accurate, reliable, and comprehensive analytical results. For example, some composite sensors simultaneously utilize optical and electrochemical signal transduction to monitor biomolecular binding events. In such systems, optical signals may report molecular interactions at the sensor interface, while electrochemical signals reflect corresponding changes in current or potential. This dual-mode output enhances detection robustness and allows operation across diverse physicochemical environments. However, for ZEN detection, the practical implementation of optical-electrochemical composite sensors remains largely conceptual. More commonly, current approaches combine multiple optical technique, such as a method integrating fluorescence with SERS reported by Wu et al. (40). This system employed upconverting nanoparticles (UCNPs) to simultaneously detect three mycotoxins, ZEN, fumonisin B1 (FB1), and ochratoxin A (OTA), demonstrating the potential of multi-signal strategies for multiplexed analysis.

In conclusion, signal transduction methods form a critical foundation of sensor technology, directly determining key performance metrics such as sensitivity, specificity, and response speed in various application scenarios. As technology advances, future sensors are expected to adopt increasingly diverse and intelligent signal transduction schemes, further driving innovation across scientific and industrial fields.

## 3 Application of optical aptasensor in ZEN detection

### 3.1 Fluorescent aptasensor

Fluorescent aptasensors are widely used for ZEN detection due to their operational simplicity, high efficiency, and excellent sensitivity. Their detection mechanisms primarily rely on fluorescence enhancement (turn-on), fluorescence quenching (turn-off), or fluorescence resonance energy transfer (FRET). In FRET-based systems, energy is transferred from an excited donor fluorophore to a nearby acceptor molecule (41). The presence of the target biomolecule alters the distance between the donor and acceptor, resulting in measurable changes in fluorescence intensity (42). A distinctive characteristic of ZEN is its ability to emit blue-green fluorescence under 360 nm UV irradiation and green fluorescence under 260 nm UV light (7).

**Figure 4:**
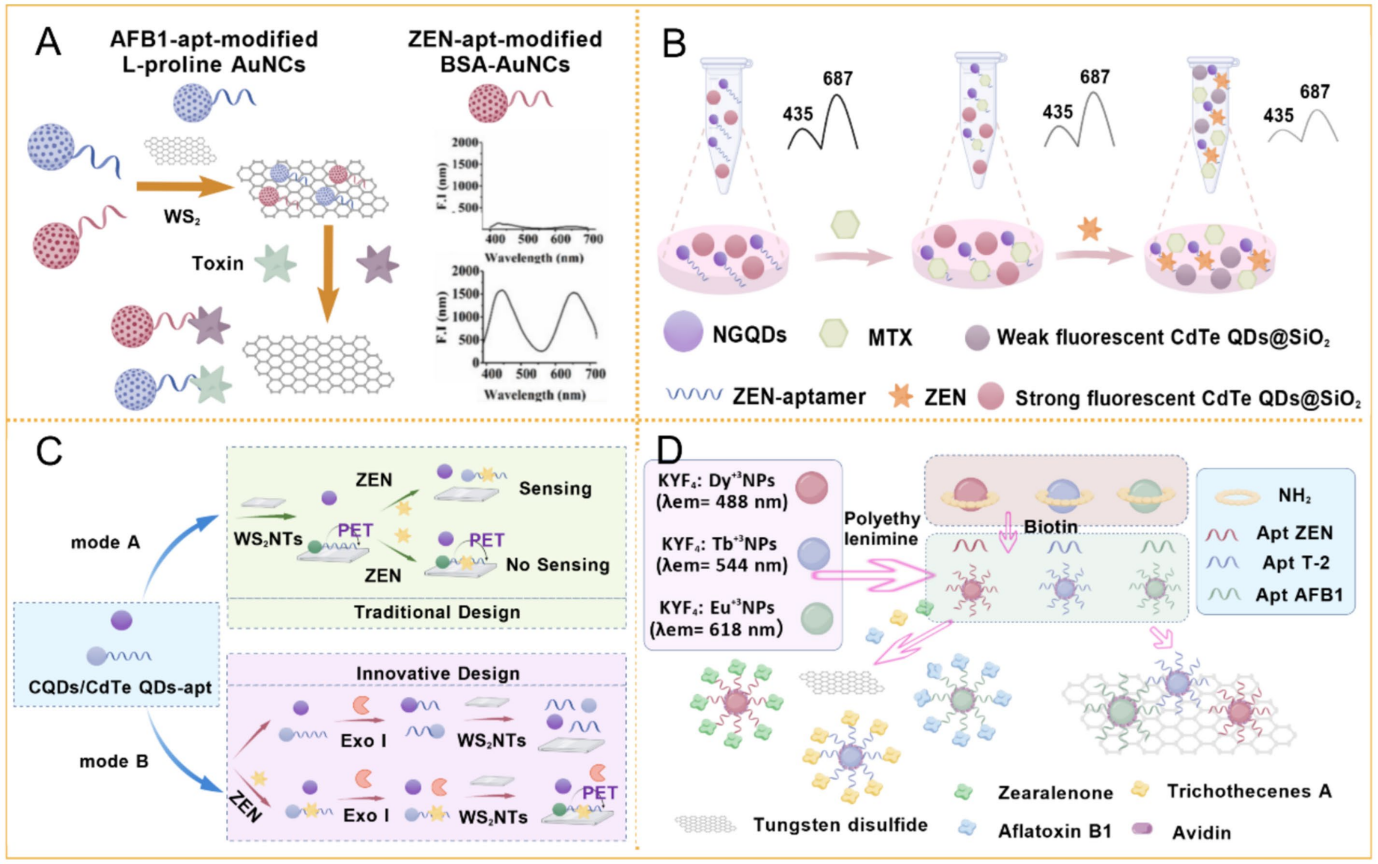
Application of fluorescent aptasensor in ZEN detection. **(A)** Aptamer induced multicolored AuNCs-WS_2_ “turn-on” FRET nano platform for dual-color simultaneous detection of AFB1 and ZEN. Reprinted from Khan et al. (44) with permission from American Chemical Society. **(B)** A “turn-on” aptasensor for simultaneous and time-resolved fluorometric determination of ZEN, trichothecenes A and aflatoxin B1 using WS_2_ as a quencher (52). **(C)** Inner filter effect-modulated ratiometric fluorescence aptasensor based on competition strategy for ZEN detection in cereal crops: using mitoxantrone as quencher of CdTe QDs@SiO2. Reprinted from Bi et al. (18) with permission from Elsevier. **(D)** A ratiometric fluorescence aptasensor based on photoinduced electron transfer from CdTe QDs to WS_2_ NTs for the sensitive detection of ZEN in cereal crops. Reprinted from Bi et al. (59) with permission from Elsevier.

With advances in nanotechnology, various types of nanomaterials are widely used in fluorescence sensing platforms due to their unique electronic and optical properties (43). For instance, Khan and Niazi’s teams developed a turn-on FRET biosensor utilizing WS_2_ nanosheets and dual-color gold nanoclusters (AuNCs) synthesized with L-proline and bovine serum albumin (44). This system enabled the simultaneous detection of ZEN and AFB1 (Figure 4A), achieving a linear range of 0.32–320 pg./mL and a detection limit (LOD) of 0.53 pg./mL for ZEN, thereby establishing a novel approach for metal nanocluster-based fluorescent switching probes. Silver nanomaterials have also been utilized in turn-on aptasensors. One such sensor employed aptamer-modified silver nanoclusters (AgNCs) and porous Fe_3_O_4_/carbon octahedra derived from metal–organic frameworks (MOFs) as a FRET donor–acceptor pair, leading to fluorescence quenching upon target binding (45). This system exhibited a linear range of 0.01–250 ng/mL and an LOD of 2 × 10^−3^ ng/mL. Similarly, Yin et al. (23) designed a FRET-based quenching system using carbon dots (CDs) and AgNPs.

In recent years, advancements in dual-mode technology have garnered significant interest among researchers. A dual-mode optical aptasensor, based on Exo I circular digestion and a cooperative enhancement strategy, combines colorimetric detection using Poly-HRP with fluorescence detection employing FAM-SGI. This sensor achieved LODs of 0.067 ng/mL and 0.009 ng/mL for the colorimetric and fluorescence modes, respectively. This innovative method holds great potential for toxin detection (46). Another novel dual-peak aptasensor, utilizing NH_2_-UiO-66 nanoparticles, also integrates fluorescence and colorimetric techniques, with LODs of 0.03 ng/mL and 1.16 ng/mL, respectively (47). Furthermore, Wu et al. (40) combined FRET with SERS, achieving a linear range of 0.1–100 ng/mL and an LOD of 0.03 ng/mL for ZEN. This platform also enabled the detection of OTA and FB1. Despite its accuracy and multiplexing capability, the method requires approximately 2 h of analysis and involves complex procedures. In contrast, a graphene oxide (GO)-based FRET sensor reduced the detection time to 60 min. By labeling ZEN and patulin (PAT) aptamers with FAM and Cy3 fluorescent probes, this system enabled simultaneous detection of both mycotoxins in traditional Chinese medicine. The LODs were 2.29 nM for PAT and 0.037 nM for ZEN, with a linear range of 1–1,024 nmol/L. This method demonstrated strong anti-interference capability, reliability, and cost-effectiveness, without requiring complex pretreatment (48).

The excellent selective absorption and fluorescence quenching properties of GO, combined with the specific recognition and programmability of DNA aptamers, have enabled the development of innovative steganographic aptasensors. For example, the fluorescence of capture probes (Cy3 and Alexa Fluor 488-labeled aptamers) was quenched by GO, allowing rapid and sensitive detection of ZEN and OTA with LODs of 1.797 ng/mL and 1.484 ng/mL, respectively (49). Another magnetic graphene oxide (MGO)-based fluorescent aptasensor exploited the weakened interaction between the fluorescent probe and MGO upon ZEN binding, leading to fluorescence recovery. This method demonstrated a linear range of 5–120 μg/L, an LOD of 2.9 μg/L, and was successfully applied to wheat and yellow wine samples (50). Beyond graphene, manganese dioxide (MnO_2_) nanosheets have emerged as promising two-dimensional nanomaterials for FRET-based sensing. They offer a high specific surface area and superior luminescence quenching efficiency compared to GO. A sensor based on the interaction between fluorophore-labeled ZEN aptamers and MnO_2_ nanosheets achieved a linear range of 1.5–10.0 ng/mL and an LOD of 0.68 ng/mL (28).

Time-resolved fluorescence (TRFL) sensors constitute another important category, based on the principle that fluorescence from certain compounds decays within microseconds. By delaying measurement until short-lived background fluorescence has subsided, TRFL enables highly sensitive detection of long-lifetime signals, effectively reducing interference from biological matrices (51). Focusing on this technology, Niazia et al. (26) developed a TRFL sensor using labeled magnetic nanoparticles (MNPs) as capture probes and TRFL nanoparticle-labeled cDNA as signal probes. This sensor achieved a linear detection range of 0.001–10 ng/mL and an LOD of 0.21 pg./mL. The following year, the same group introduced a turn-on TRFL sensor that employed multi-color-emitting Dy^3+^, Tb^3+^ and Eu^3+^ nanoparticles as probes, with WS_2_ nanosheets serving as quenchers. This system detected ZEN with an LOD of 0.51 pg./mL and a linear range of 0.001–100 ng/mL, enabling simultaneous detection of three mycotoxins within 1 h (52) (Figure 4B).

In 2022, a three-dimensional DNA tweezer-based aptasensor was reported, achieving an LOD of 0.037 ng/mL for ZEN. This single-step assay allowed simultaneous detection of ZEN and OTA within one hour, demonstrating high specificity (53). In 2023, Ma et al. (54) constructed a fluorescence polarization sensor employing molecular dynamics, circular dichroism, and isothermal titration calorimetry, achieving an LOD of 0.004 ng/mL and a linear range of 0.01–100 ng/mL within 1 h. Another system utilizing CRISPR-Cas12a and Nt. AlwI for signal amplification achieved an LOD of 0.213 pg./mL within a linear range of 1–1,000 pg./mL, albeit with a longer detection time of two hours (55). Recently, a simple and efficient CRISPR/Cas12a-assisted fluorescence aptasensor was developed for the simultaneous detection of ZEN and OTA. This method demonstrated strong detection capabilities under optimized conditions, with LODs of 190 pM and 931 pM, respectively (56). Compared to CRISPR-Cas, Argonaute (Ago) offers precise endonuclease activity on any target sequence and can utilize both RNA and DNA as guide molecules. The method for detecting ZEN using rolling circle amplification (RCA) combined with the pfAgo protein achieved an LOD of 5.3 pg./mL within the range of 0.01–10 ng/mL (57).

Quantum dots (QDs) have garnered significant attention in the design of fluorescent aptasensors. In 2020, a single-wavelength-excited dichroic fluorescent nanoprobe was developed using QDs with red and green emission serving as reference and response signals, respectively. This method demonstrated an LOD of 7.5 nM, a linear range of 31.4–628 nM, and exhibited high stability and selectivity (58). The following year, Bi’s team created a ratiometric sensor employing ZEN aptamer-modified nitrogen-doped graphene quantum dots (NGQDs-apt) alongside CdTe QDs encapsulated in silica spheres (CdTe QDs@SiO_2_) to detect ZEN in corn and barley flour. The sensor achieved a linear range of 0.32–320 pg./mL and an LOD of 0.32 pg./mL (18) (Figure 4C). Subsequent research by the same group investigated semiconductor QDs combined with WS_2_ nanosheets for ZEN detection in rice and corn flour, achieving an LOD of 0.1 pg./mL and a linear range of 1–50 pg./mL. This study also represented the first application of WS_2_ nanotubes to quench the fluorescence of CdTe QDs-apt (59) (Figure 4D). Na et al. (60) designed a rapid and sensitive switchable fluorescent aptasensor based on oxidized single-walled carbon nanohorns (oxSWCNHs) and nitrogen-doped carbon dots (NCDs-apt). The oxSWCNHs, recognized for their ultra-lightweight nature, high thermal and mechanical stability, and large surface area, functioned as efficient fluorescence quenchers. Under optimized conditions, the sensor achieved an LOD of 18 ng/mL and a linear range of 20–100 ng/mL.

A portable aptasensor utilizing streptavidin-modified magnetic microspheres (MMPs) and a hybridization chain reaction (HCR) enabled the simultaneous detection of ZEN and T-2 toxin in corn and oat flour. The linear detection ranges were 0.01–100 ng/mL for ZEN and 0.001–10 ng/mL for T-2, with LODs of 1.2 pg./mL and 0.1 pg./mL, respectively (61). In 2020, a sensor incorporating gold nanorods (AuNRs), UCNPs, fluorescent dyes, and DNA sequences achieved simultaneous detection of ZEN and FB1 with LODs of 0.01 μg/L and 0.003 ng/L, respectively (62). In the same year, a green enzyme-linked immunosorbent assay (ELISA) based on a single-stranded binding protein (SSB) and a co-aptamer strategy was reported. By leveraging the high catalytic activity of the biotin-streptavidin system and horseradish peroxidase (HRP), this method achieved high specificity and an LOD of 377 ng/L (63). Additionally, a fluorescent sensor based on mesoporous silica nanoparticles demonstrated a linear range of 0.005–150 ng/mL and an LOD of 0.012 ng/mL. This system was successfully applied to ZEN detection in grains and grain-derived products, showing satisfactory recovery rates (64).

In summary, the integration of advanced materials such as metal nanomaterials, MOFs, QDs, and TRFL technologies has significantly enhanced the performance of fluorescent aptasensors. These systems demonstrate high sensitivity, making them suitable for detecting trace levels of toxins. TRFL and GO-based FRET sensors enable rapid detection within 1 h, while GO and MnO_2_ nanosheets provide wide linear detection ranges. Although MnO_2_ nanosheets offer a larger surface area and higher quenching efficiency, GO remains more attractive due to its lower cost. Future developments should focus on designing low-cost, portable sensors to better support food safety monitoring.

### 3.2 Colorimetric aptasensor

**Figure 5:**
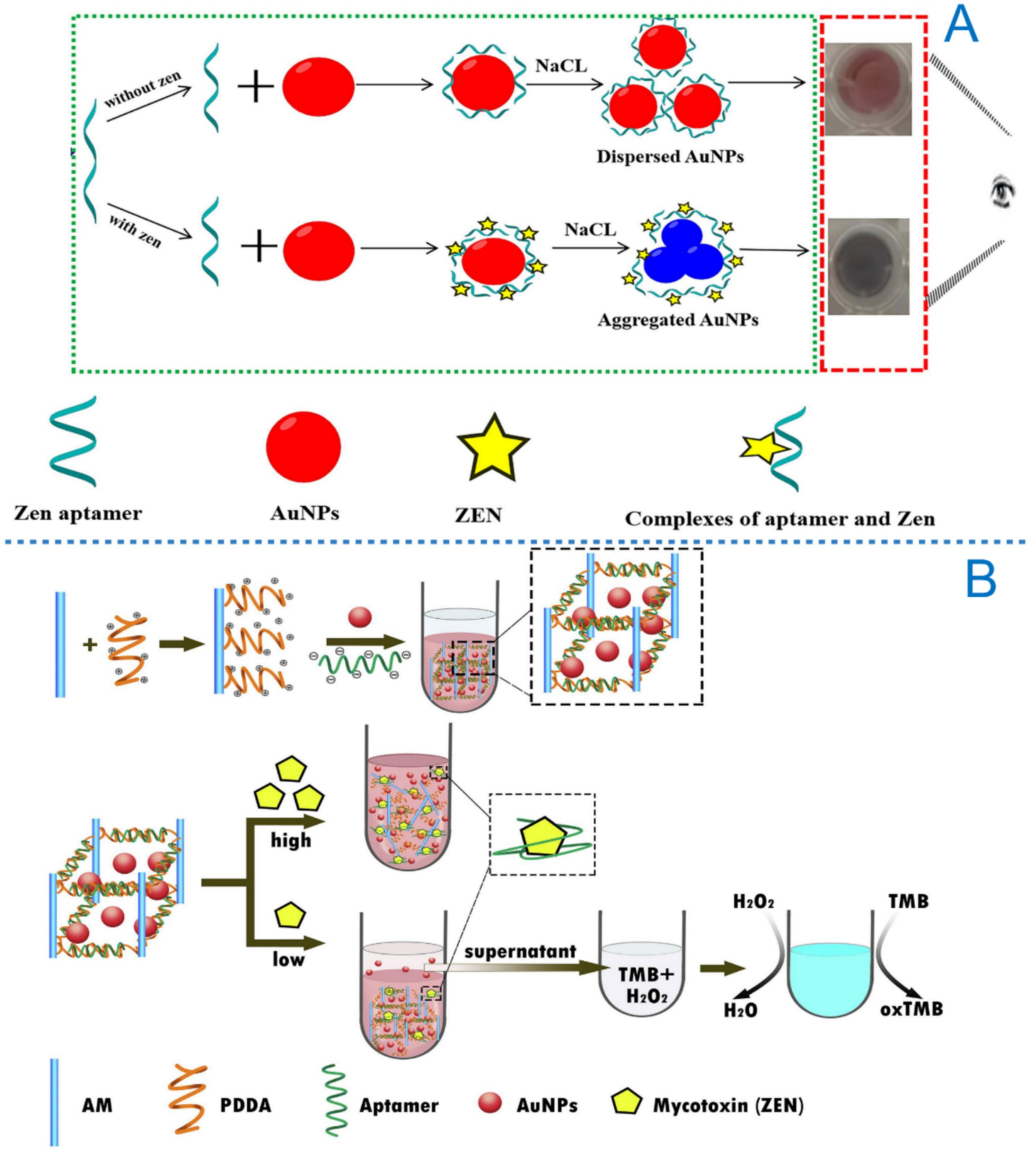
Application of colorimetric aptasensor in ZEN detection. **(A)** A smartphone-assisted colorimetric aptasensor based on aptamer and gold nanoparticles for visual, fast and sensitive detection of ZEN in maize. Reprinted from Zhang et al. (70), licensed under CC BY-NC-ND 4.0. **(B)** A label-free visual aptasensor for ZEN detection based on target-responsive aptamer-cross-linked hydrogel and color change of gold nanoparticles. Reprinted from Liu et al. (71) with permission from Elsevier.

Colorimetric aptasensors are distinguished by their cost-effectiveness, high sensitivity, strong specificity, and visual readability. In recent years, the integration of advanced nanomaterials has significantly propelled the development of these sensors (31). AuNPs in particular, are widely utilized due to their high extinction coefficient and pronounced catalytic activity in the visible light region (65). A visual colorimetric method developed by Sun et al. (66) exploited the inhibitory effect of ZEN aptamers on the peroxidase-mimicking activity of AuNPs. As the concentration of ZEN varied, the solution color changed correspondingly. Under optimized conditions, this method achieved a detection range of 10–250 ng/mL and an LOD of 10 ng/mL. While this approach allowed for naked-eye observation and exhibits high specificity, its sensitivity still requires further improvement. In the same year, another AuNPs-based colorimetric sensor was reported, incorporating off-target-induced strand displacement, Exonuclease III (Exo III) for signal amplification, and 4-nitrophenol as a colorimetric substrate. This system detected ZEN across a wide linear dynamic range of 20–80,000 ng/L, with an LOD of 10 ng/L (67). Furthermore, Zhang et al. (68) introduced a smartphone-assisted colorimetric aptasensor using AuNPs as indicators. This method achieved an LOD of 5 ng/mL and a linear range of 5–300 ng/mL, demonstrating high specificity and stability. By relying solely on a smartphone for signal readout, the assay requires no expensive instrumentation, is simple to operate, and can be completed within 15 min, highlighting its strong potential for practical applications (Figure 5A). Recently, a new type of colorimetric sensor was developed that leverages the synergistic interaction between Au/CoOOH and Au nanoparticles. This interaction enhanced the catalytic efficiency of CoOOH by 3.5 times, achieving an LOD of 0.23 ng/mL (69). In the same year, Wang et al. (70) synthesized another highly sensitive colorimetric aptasensor for ZEN detection using a self-assembly strategy mediated by AuNPs. By adjusting the amount of AuNPs, they promoted the rational design of core-shell nanomaterials for biosensing applications.

Another innovative approach combines AuNPs with hydrogels (71). In the presence of ZEN, aptamer-target binding triggers the dissociation of the hydrogel, releasing encapsulated AuNPs into the supernatant and producing a visible color change. This system enables low-dose visual detection, with absorbance showing a linear relationship to ZEN concentration between 2.5–100 ng/mL and an LOD of 0.98 ng/mL in real samples such as corn and beer (Figure 5B). Also employing hydrogel technology, Sun et al. developed a colorimetric filter sensor by integrating a MOF nanozyme with hyaluronic acid (HA)-DNA hydrogel. This sensor exhibited excellent catalytic performance and stability, with a linear range of 0.001–200 ng/mL and an LOD of 0.8 pg./mL. The total assay time, including sample preparation, was under 45 min (72).

Nanozyme-based colorimetric strategies have also attracted significant attention. Notably, the electrochemical-colorimetric dual-mode detection of ZEN, based on the DNA walking system driven by restriction endonucleases, offers the advantages of dual signal amplification and mutual verification of dual modes (73). The detection limits of the colorimetric and the electrochemical methods are 3.44 × 10^−10^ mol/L and 3.39 × 10^−9^ mol/L, respectively. While many redox-based nanozyme sensors are susceptible to interference from endogenous reducing agents in complex samples, Xiong et al. (74) designed a non-redox colorimetric sensor utilizing the intrinsic phosphatase-like activity of CeO_2_ nanoparticles. This system effectively avoided interference from reducing agents, opening new avenues for food safety and biological detection.

Another innovative colorimetric aptasensor utilizes a multivalent aptamer scaffold combined with a light-responsive nanozyme that employs dissolved oxygen as the electron acceptor instead of H_2_O_2_. The reaction progress can be conveniently controlled by switching the light on or off, offering a simple, sensitive, and instrument-free detection platform with significant potential for monitoring small-molecule hazards (75). Additionally, a highly efficient ZEN colorimetric aptasensor was constructed using stimulus-responsive aptamer-functionalized MOF nanocapsules and a trivalent DNA peroxidase mimic. When applied to corn and wheat samples, this sensor demonstrated a linear detection range of 0.01–100 ng/mL and an impressive LOD of 0.36 pg./mL (76).

Despite advantages such as simplicity, low cost, and visual detection, colorimetric methods often perform optimally only in controlled buffer systems. In real-sample analyses, environmental interferences can compromise accuracy and reliability. Furthermore, the success rate of generating high-affinity aptamers remains relatively low. Nevertheless, ongoing advances in nanotechnology have facilitated the widespread use of novel materials, including UCNPs, QDs, and MOFs, as signal probes or sensing platforms in aptasensor design (77). In particular, plasmonic nanomaterials such as AuNPs continue to be favored in colorimetric biosensing due to their strong optical properties and ability to produce visually distinguishable color changes. It is anticipated that continued technological innovation will address current limitations in on-site applicability, enabling colorimetric aptasensors to realize their full potential in practical food safety monitoring.

### 3.3 SERS aptasensor

SERS has emerged as a rapid and label-free analytical technique for the determination of ZEN. Owing to its operational simplicity, cost-effectiveness, rapid analysis, and ability to provide unique spectral fingerprints, SERS enables highly sensitive and high-resolution detection of ZEN without the need for complex sample pretreatment. These characteristics make it well-suited for quantitative analysis and real-time monitoring, offering an efficient approach for ZEN detection (78).

**Figure 6:**
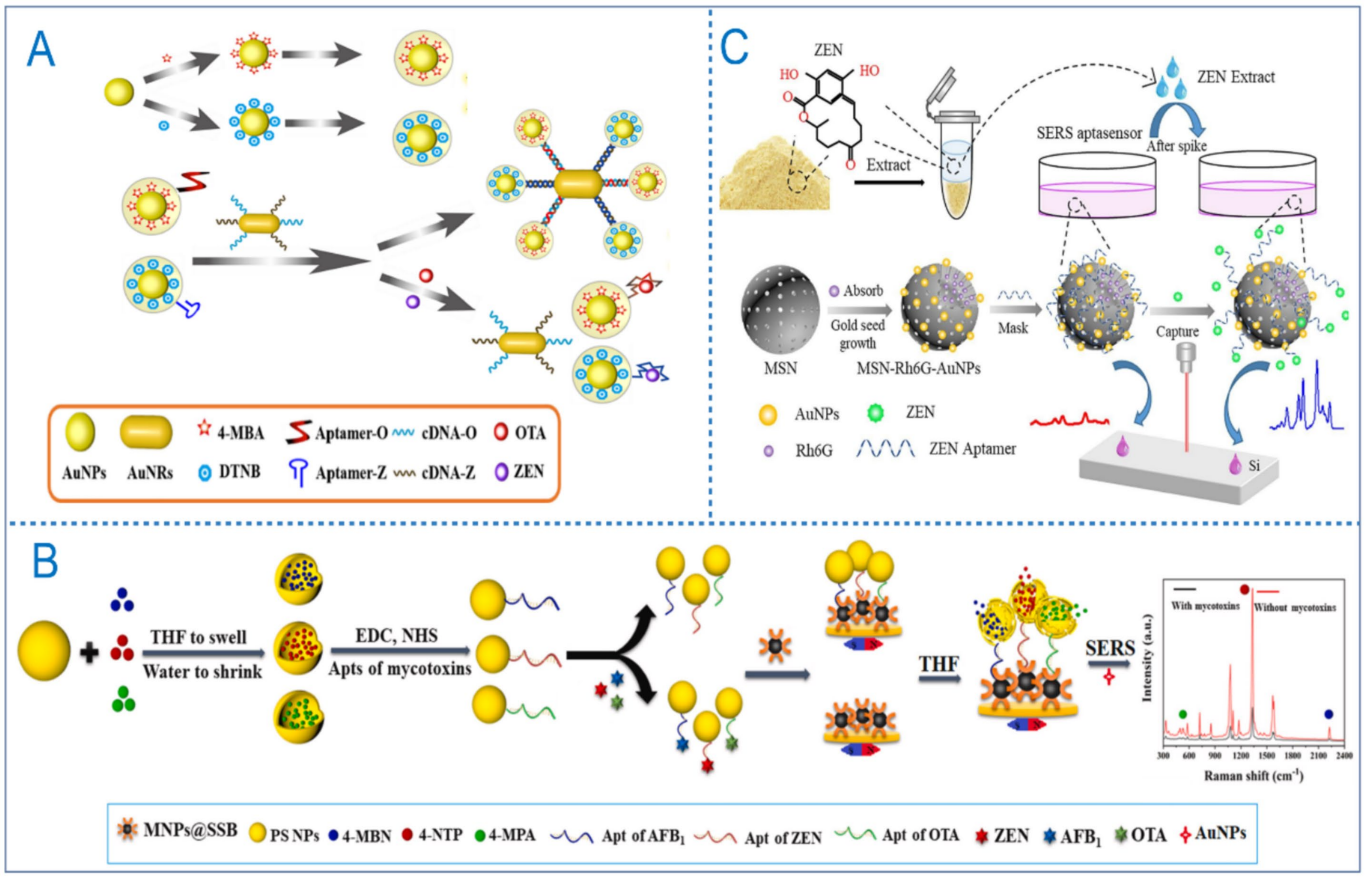
Application of SERS aptasensor in ZEN detection. **(A)** Surface-enhanced Raman spectroscopy aptasensor for simultaneous determination of OTA and ZEN using Au@Ag core-shell nanoparticles and gold nanorods. Reprinted from Chen et al. (79) with permission from Springer Nature. **(B)** Low background interference SERS aptasensor for highly sensitive multiplex mycotoxin detection based on polystyrene microspheres-mediated controlled release of Raman reporters. Reprinted from Yang et al. (81) with permission from Elsevier. **(C)** Novel mesoporous silica surface loaded gold nanocomposites SERS aptasensor for sensitive detection of ZEN. Reprinted from Guo et al. (84) with permission from Elsevier.

One representative SERS aptasensor employed thiolated complementary DNA (SH-cDNA) modified gold nanorods as capture probes and SH-aptamer-modified Au@Ag core–shell nanoparticles as reporting probes for the simultaneous detection of ZEN and OTA. Upon hybridization between the aptamer and its complementary strand, the reporting probe generated a strong SERS signal. For ZEN, the sensor exhibited a linear range of 0.05–500 ng/mL and a detection limit of 0.054 ng/mL, demonstrating excellent performance (Figure 6A) (79). Another SERS biosensor also utilized Au@Ag core–shell nanoparticles as reporting probes, combined with thiolated ZEN aptamer-complementary DNA modified on Fe_3_O_4_@Au core–shell structures as capture probes. This configuration achieved a wide linear range of 0.005–500 ng/mL and an LOD of 0.001 ng/mL for ZEN (80).

Based on FRET and SERS technologies, a sensing platform capable of simultaneously detecting three mycotoxins, including ZEN, OTA and FB1, has been reported, with detection limits of 0.03 ng/mL, 0.01 ng/mL and 0.02 pg./mL, respectively (40). This multiplex assay demonstrated high selectivity, highlighting its potential for multi-channel mycotoxin analysis. However, the method requires approximately 2 h for analysis and involves relatively complex procedures, indicating a need for further optimization. Additionally, a SERS aptasensor utilizing polystyrene (PS)-assisted signal amplification has been developed for the simultaneous detection of multiple mycotoxins (81). Due to the high loading capacity of PS, this system achieved exceptionally low LOD of 0.159 fg/L for ZEN, with corresponding values of 2.015 fg/L and 1.561 fg/L for OTA and AFB1, respectively (Figure 6B). This approach provides a novel design concept for future Raman-based detection strategies.

While most reported aptasensors are limited to the detection of a single mycotoxin, real-world food samples often contain multiple co-occurring toxins. In this context, SERS-based multi-target detection holds considerable practical relevance (82, 83). For instance, a SERS aptasensor fabricated using mesoporous silica-loaded gold nanocomposites demonstrated significant potential for ZEN detection in maize (84). The small nanogaps between gold nanoparticles enabled the MSN-Rh6G-AuNP composite to exhibit strong SERS performance under laser excitation. The sensor showed a linear response to ZEN within the range of 3–200 ng/mL and an LOD of 0.0064 ng/mL (Figure 6C).

In summary, SERS represents a rapid, label-free, and efficient tool for ZEN detection. Its advantages include ease of use, low cost, rapid analysis, and high resolution, making it suitable for quantitative and real-time monitoring applications. By incorporating various plasmonic probes such as AuNRs and Au@Ag core–shell nanoparticles, SERS aptasensors can achieve nanogram-level detection of ZEN as well as other mycotoxins like OTA. Integrated platforms combining FRET and SERS further enable the simultaneous detection of ZEN, OTA, and FB1 with high selectivity. Although challenges such as lengthy analysis times and procedural complexity remain, SERS aptasensors show great promise for multi-target mycotoxin detection in complex food matrices (Table 1).

**Table 1:** Optical aptasensors for detection of ZEN.

Method	Materials	Commodity	Working range (ng/mL)	LOD (ng/mL)	Recovery	References
Fluorescent	Lp-AuNCs·BSA-AuNCs·WS_2_	Maize	3.2 × 10^−4^–3.2 × 10^−1^	5.3 × 10^−4^	94.36–110.40	(44)
Fluorescent	AgNCs·Fe_3_O_4_/Carbon octahedron	Maize, Wheat	0.01–250	2 × 10^−3^	94.2–102.0	(45)
Fluorescent	CHA·Ag NCs	Maize, Beer	1.3 × 10^−3^–100	3.2 × 10^−4^	96.9–107.8	(23)
Dual-mode	Exo I	–	–	0.067/9 × 10^−3^	–	(46)
dual-mode	NH_2_-UiO-66@MB	Cereal	0.125–200/2.5–300	0.03/1.16	97.43–103.71	(47)
Dual-mode	UCNP·AuNP	Maize	0.1–100	0.03	90–107	(40)
Fluorescent	GO·Cy3	TCM	3.18 × 10^−4^–3.26 × 10^−1^	1.18 × 10^−5^	102.8–112.7	(48)
Fluorescent	Cy3 aptamer and Alexa Fluor 488 aptamer	Wine	–	1.797	89–99.89	(49)
Fluorescent	MGO	Wheat, Rice wine	5–120	2. 9	76.4–118.8	(50)
Fluorescent	MnO_2_·6-FAM	Maize	1. 5–10.0	0.68	93.8–102.6	(28)
Fluorescent	MNPs	Maize, Wheat	1 × 10^−3^–10	2.1 × 10^−4^	90.04–114.75	(26)
Fluorescent	Dy^3+^, Tb^3+^, Eu^3+^·WS_2_	Maize	1 × 10^−3^–100	5.1 × 10^−4^	94.4–98.0	(52)
Fluorescent	DNA tweezers	Maize, Wheat	0.5–50	0.037	95.8–110.2	(53)
Fluorescent	Z19-ZEN	Corn, Wheat	0.01–100	4 × 10^−3^	–	(54)
Fluorescent	CRISPR-Cas12a, Nt. AlwI	Corn oil	1 × 10^−3^–1	2.13 × 10^−4^	94.18–106	(55)
Fluorescent	CRISPR/Cas12a	Maize oil	7.95 × 10^−2^–81.4	6.04 × 10^−2^	89.17–109.88	(56)
Fluorescent	pfAgo	Corn flour	0.01–10	5.3 × 10^−3^	104.58–112.82	(57)
Fluorescent	CdTe quantum dots	Corn flour, Wheat flour	9.99–200	2.39	92.20–99.98	(58)
Fluorescent	NGQDs-apt·CdTe QDs@SiO_2_	Corn flour, Barley flour	3.2 × 10^−4^–3.2 × 10^−1^	3.2 × 10^−4^	96.9–107.8	(18)
Fluorescent	QDs·WS_2_ NTs	Rice flour, Corn flour	1 × 10^−3^–5 × 10^−2^	1 × 10^−4^	95.6–105	(59)
Fluorescent	NCDs-apt·oxSWCNHs	Corn flour	20–100	18	99.5–114.3	(60)
Fluorescent	MMPs and HCR	Spiked corn, Oat flour	0.01–100	1.2 × 10^−3^	90.7–111.10	(61)
Fluorescent	AuNR·UCNP	Maize	0.05–100	0.01	89.9–106.6	(62)
Fluorescent	SSB·SA·HRP	Corn	–	0.377	83.36–125.28	(63)
Fluorescent	MSNs-NH_2_·aptamer- fam	Grain, Cereal	0.005–150	0.012	83.3–101.5	(64)
Fluorescent	CdTe QDs. AuNP	Corn, Soybeans, wheat	0.01–100	3.5 × 10^−3^	96.5–104.0	(127)
Fluorescent	Cu/UiO-66	Cereal	0.5–60	0.048	83.67–106.8	(128)
Fluorescent	UiO-66-NH_2_ MOF	Wheat, Corn flour	0.01–100	5 × 10^−3^	85–114	(129)
Fluorescent	N-CDs	Corn flour, Corn oil	0.25–200	0.0875	84.7–108.6	(130)
Fluorescent	TdT	–	0.1–1,000	0.1	–	(131)
Colorimetric	AuNPs	Maize, Corn oil	10–250	10	92.0–110.0	(65)
Colorimetric	AuNPs·Exo III·4-nitrophenol	Human serum	0.02–80	0.01	95.0–103.0	(66)
Colorimetric	AuNPs · ZEN aptamer	Maize	5–300	5	81.3–96.4	(67)
Colorimetric	Au/CoOOH	Cereal	0.3–500	0.23	90.65–105.63	(68)
Colorimetric	Au@Cu_2_O nanoparticles	Corn flour, wheat flour	5 × 10^−4^– 5	2.4 × 10^−4^	92.7–105	(69)
Colorimetric	target-responsive aptamer-cross-linked hydrogel	Maize, Beer	2.5–100	0.98	98.8–101.5	(70)
Colorimetric	MOFzyme·HA-DNA hydrogel	Maize, Soya	1 × 10^−3^–200	8 × 10^−4^	94.0–109.0	(71)
Dual-mode	SA-MBs	Corn flour	3.18 × 10^−4^–3.18 × 10^−1^/3.18 × 10^−3^–3.18	1.09 × 10^−4^/1.078 × 10^−3^	92.1–105.9	(73)
Colorimetric	CeO_2_	-	3.18 × 10^3^–6.36 × 10^4^	6.36	–	(74)
Colorimetric	Polyvalent aptamer scaffold light-responsive oxidase-like nanozyme	Corn, Wheat	0.01–1	8.7 × 10^−3^	92.0–111.0	(75)
Colorimetric	MOF·DNAzyme	Maize, Wheat	0.01–100	3.6 × 10^−4^	94.6–108.7	(76)
SERS	Au@Ag ·AuNRs	Corn, Wheat	0.05–500	0.054	96.0–110.7	(79)
SERS	Fe_3_O_4_@Au·Au@Ag	Beer, Wine	0.005–500	0.001	96.0–111.4	(80)
SERS	UCNP·AuNP	Corn	0.1–100	0.03	90–107	(40)
SERS	PS·MNPs@SSB	Maize	2.54 × 10^−3^–3.18	5.06 × 10^−8^	85.4–112.1	(81)
SERS	MSN-Rh_6_GAuNPs	Corn	3–200	6.4 × 10^−3^	94.68–118.58	(84)

## 4 Application of electrochemical aptasensor in ZEN detection

Electrochemical sensors are extensively utilized in biological, environmental, industrial, and pharmacological research due to their reliability, high sensitivity, accuracy, low cost, and rapid response. Specifically, in the detection of ZEN electrochemical aptasensors typically demonstrate greater sensitivity than other sensing platforms. Recent advancements in this area are summarized in Table 2.

**Table 2:** Electrochemical aptasensors for detection of ZEN.

Method	Materials	Commodity	Working range (ng/mL)	LOD (ng/mL)	Recovery	References
EC	MXene/PEDOT: PSS	Maize	1 × 10^−3^–100	2.8 × 10^−5^	99.63–100.34	(85)
EC	Ferro/ferricyanide	Corn	0.01–1,000	0.017	87.0–110.0	(86)
EC	PEI-MoS_2_-MWCNTs	Beer	5 × 10^−4^–50	1.7 × 10^−4^	–	(87)
EC	Thi·hcPt@AuNFs/PEI-rGO	Maize	5 × 10^−4^–500	1.7 × 10^−4^	97.3–106.0	(88)
EC	Au/4-MP	Corn, wheat	0.1–250	0.182	95–105%	(89)
EC	hcPt@AuNFs/PEI-rGO·Fe_3_O_4_ NRs/rGO	Maize	0.5–50	1.05 × 10^−4^	91.6–104.4	(90)
EC	CS@AB-MWCNTs	Corn oil, Corn flour	1 × 10^−5^–1	3.64 × 10^−6^	92.81–99.58	(91)
EC	Au@Pt/Fe-N-C · IMB/IAg^+^	Corn flour	1 × 10^−5^–10	5 × 10^−6^	89.7–101.2	(92)
EC	Ce_3_NbO_7_/CeO_2_ @Au·PdNi@MnO_2_/MB	Human serum	15.9–4,134	4.04	90.0–112.0	(93)
EC	RecJf Exo·	Corn flour, Beer	5 × 10^−6^–50	5.1 × 10	86.0–111.0	(94)
EC	Exo I·bHCR	Corn powder	3.18 × 10^−3^–318	9.86 × 10^−4^	83.4–97.5	(95)
EC	Thi · FC_6_S · rMoS_2_-Au	Maize	1 × 10^−3^–10	5 × 10^−4^	95.9–105.2	(96)
EC	MTV polyMOF(Ti)·	Maize, Peanut, Beer	1 × 10^−5^–10	7 × 10^−6^	96.50–104.47	(97)
EC	40-mer ZEA aptamer	–	1 × 10^−3^–100	1.5 × 10^−3^	–	(98)
EC	Ce-TpBpy COF·Au NPs@Ce-TpBpy	Corn flour	1 × 10^−3^–10	3.89 × 10^−4^	93.0–104.7	(99)
EC	PDANSs·	Corn flour	1 × 10^−3^–0.15	1.8 × 10^−4^	98–108	(100)
EC	CRISPR/Cas12a	Corn flour	1 × 10^−5^–10	6.27 × 10^−6^	93.89–107.33	(132)
EC	SA-MBs	Corn flour	3.18 × 10^−2^–318	1.40 × 10^−2^	99.4–109.5	(133)
EC	Co_3_O_4_/MoS_2_/Au	Corn flour	3.18 × 10^−2^–318	2.70 × 10^−2^	94.8–105.0	(134)
EC	Cu-Zr, MOF-818	Corn, Wheat	1 × 10^−4^–10	2.8 × 10^−5^	96.5–105.0	(135)
EC	CuBi-BPDC	Milk, Rice	1 × 10^−6^–10	1.9 × 10^−7^	92.0–109.9	(124)
EC	P-Ce-MOF@MWCNTs	semen coicis	5 × 10^−5^–50.0	1 × 10^−5^	97.0–103	(136)
EC	Ti_3_C_2_Tx MXene	Corn flour, Beer	1 × 10^−5^–10	1.64 × 10^−6^	87.7–105.2	(137)
ECL	Au NPs@PBAs	Corn flour	5 × 10^−4^–100	3.7 × 10^−4^	–	(138)
ECL	TPE NA	Corn, Wheat flour	1 × 10^−6^– 00	3.62 × 10^−7^	95.9–113.0	(139)
ECL	Ru(bpy)_3_SiO_2_ NPs·NGQDs	Corn flour	1 × 10^−5^–10	1 × 10^−6^	92.2–111.1	(102)
ECL	MB, Ru(bpy)_3_^2+^	Corn	1 × 10^−6^–50	8.5 × 10^−7^	93.2–102.8	(103)
ECL	SnS_2_ QDs/g-C_3_N_4_·CuO/NH_2_-UiO-66	Corn juice	1 × 10^−7^–500	8.5 × 10^−5^	96.5–104.8	(104)
PEC	ZnO-NGQDs	Corn flour, Rice flour, Barley flour	1 × 10^−4^–100	3.3 × 10^−5^	92.9–107.2	(105)
PEC	Cu–N_2_ single-atom	Flour	1 × 10^−4^–20	2.4 × 10^−5^	94.8–110.0	(106)
PEC	COF-367 NSs/CdS QDs/Ag_2_S	Corn	1.59 × 10^−3^–31.8	3.50 × 10^−4^	98.7–102.7	(107)
PEC	NiTiO_3_/CdIn_2_S_4_ *Z*-scheme heterojunction	Corn, Wheat	1 × 10^−4^–1,000	2.6 × 10	97.6–98.0	(108)
PEC	CdS/ethylenediamine 3D nanowires network	–	1 × 10^−4^–100	2.4 × 10^−5^	–	(109)
PEC	Z-type BiOI/g-C_3_N_4_@Au/Bi_2_S_3_ heterojunctions	–	1 × 10^−7^–1.0	8.7 × 10^−8^	–	(110)
PEC	Cu_2_MoS_4_/CdS/In_2_S_3_ nanoclusters	Rice, Beer	1 × 10^−5^– 1,000	2.38 × 10^−6^	97.66–106.15	(111)

### 4.1 EC aptasensor

A groundbreaking approach incorporated MXene/PEDOT: PSS nanocomposites into an unlabeled aptamer sensing platform for ZEN detection. This innovative sensor successfully detected ZEN over a concentration range of 1 pg./mL to 100 ng/mL, demonstrating an LOD of 0.028 pg./mL (85), thus showcasing the potential of composite materials in improving detection limits. Similarly, another unlabeled competitive aptasensor utilized a ferro/ferrocyanide system as a redox probe for square wave voltammetry and achieved an LOD of 0.017 ng/mL under optimal conditions. This sensor operated effectively across a concentration range of 0.01–1,000 ng/mL (86), emphasizing the versatility of electrochemical methods for ZEN detection.

Several studies have focused on signal enhancement strategies to improve the performance of electrochemical aptasensors. An aptasensor utilizing a PEI-MoS_2_-MWCNTs nanocomposite material demonstrated an impressive detection performance, with a concentration range of 0.5 pg./mL to 50 ng/mL and an LOD of 0.17 pg./mL (87). Another voltammetric aptasensor developed with porous platinum nanotubes and gold nanoparticles achieved a linear range of 0.5 pg./mL to 0.5 μg/mL, reaching an LOD of 0.17 pg./mL, which was significantly lower than previous methods (88).

Moreover, advancements continued with the development of a quartz crystal microbalance (QCM) aptasensor, which utilized gold nanoparticles and electrochemically deposited diazo derivative films to amplify signals. This system exhibited a linear detection range of 0.1–250 ng/mL with an LOD of 0.182 ng/mL (89). The high sensitivity of these sensors is often attributed to efficient electron transfer between graphene surfaces and biomolecules, as highlighted by a “signal on” electrochemical sensor utilizing hollow cubic Pt@Au nanoframes, achieving a linear range of 0.5 pg./mL to 50 ng/mL and an LOD of 0.105 pg./mL (90).

**Figure 7:**
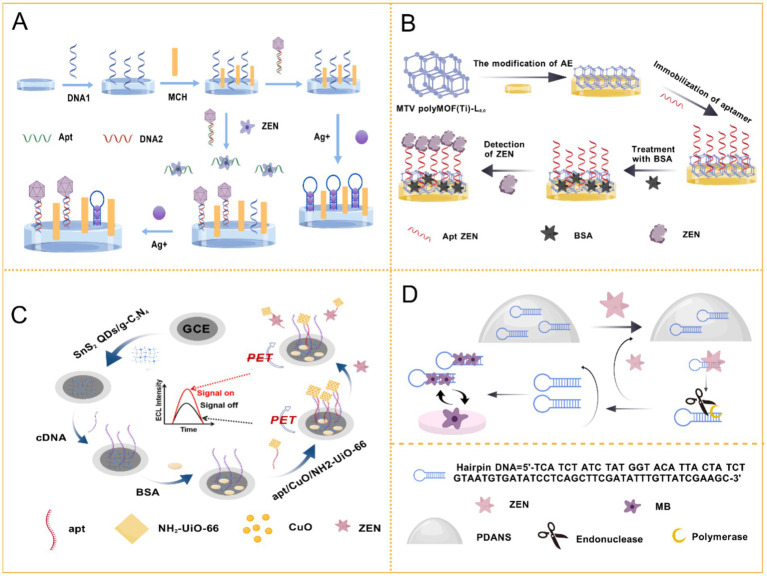
Application of electrochemical aptasensor in ZEN detection. **(A)** A methylene blue and Ag + ratiometric electrochemical aptasensor based on Au@Pt/Fe-N-C signal amplification strategy for ZEN detection. Reprinted from Suo et al. (92) with permission from Elsevier. **(B)** Electrochemical aptasensing strategy based on a multivariate polymertitanium-metal–organic framework for ZEN analysis. Reprinted from Duan et al. (97) with permission from Elsevier. **(C)** Novel ultralow-potential electrochemiluminescence aptasensor for the highly sensitive detection of ZEN using a resonance energy transfer system. Reprinted from Zhu et al. (100) with permission from the Royal Society of Chemistry. **(D)** An immobilization-free electrochemical aptamer-based assay for ZEN based on target-triggered dissociation of DNA from polydopamine nanospheres with strand displacement amplification. Reprinted from Xiang et al. (104) with permission from American Chemical Society.

Incorporating target-induced signal amplification into sensing strategies has proven effective. One approach utilized carboxy-functionalized carbon black labeled with ZEN-specific aptamers, resulting in an LOD of 3.64 fg/mL in corn oil and corn meal (91). The introduction of dual-signal probes such as methylene blue (MB) and Ag^+^ also demonstrated substantial signal amplification through Au@Pt/Fe–N–C nanocomposites, achieving a linear relationship between 1 × 10^−5^ and 10 ng/mL with an LOD of 5 fg/mL (92) (Figure 7A). A linear relationship was observed between 1 × 10^−5^ and 10 ng/mL, with an LOD of 5 fg/mL. Yan’s team also utilized MB in an electrochemical aptasensor incorporating Nb-based materials. The system employed Ce_3_NbO_7_/CeO_2_@Au hollow nanospheres as the electrode modification material and PdNi@MnO_2_/MB as the signal tag, taking advantage of the high surface area, conductivity, stability, and abundant binding sites of the nanocomposite (93). Both of these electrochemical sensors demonstrated high accuracy, reliability, and application potential.

Exonuclease-assisted amplification techniques have emerged as powerful tools for enhancing detection sensitivity. A homogeneous electrochemical aptasensor demonstrated the current difference in the presence and absence of ZEN, utilizing RecJf exonuclease for aptamer cleavage. This sensor detected ZEN in corn flour and beer over a linear range of 5 fg/mL to 50 ng/mL with an LOD of 0.51 fg/mL (94). Subsequently, Liao et al. developed an ultrasensitive sensing strategy employing exonuclease I with a branched hybridization chain reaction, achieving an impressive LOD of 3.1 × 10^−12^ mol/L within a linear range of 10^−11^ to 10^−6^ mol/L (95).

The incorporation of metal nanoparticles, known for their unique properties, has been pivotal in enhancing sensor performance. For instance, Han’s team developed a co-reduced MoS₂ and AuNP platform, allowing simultaneous detection of ZEN and FB1, with high sensitivity and selectivity (96). Additionally, a novel multi-element titanium-based MOF achieved an impressively low LOD in various samples, spanning a detection range of 10 fg/mL to 10 ng/mL (97) (Figure 7B).

The trend toward commercialization has seen the development of portable systems. In 2021, Azri et al. (98) demonstrated that a truncated aptamer could enhance biosensor sensitivity, achieving a linear range of 0.01 to 1,000 ng/mL and an LOD of 1.5 pg./mL. The pursuit of portability culminated in 2022 with a USB-drive-based electrochemical workstation, integrated with a screen-printed electrode (SPE), exhibiting high sensitivity and an LOD of 0.389 pg./mL within a linear range of 1 pg./mL to 10 ng/mL (99). More recently, Zhu’s team developed an immobilization-free electrochemical sensor using a multifunctional hairpin DNA design, achieving an LOD of 0.18 pg./mL and applicability in corn flour samples across a detection range of 1–150 pg./mL (100) (Figure 7C).

Building upon these advancements, the future development of ZEN aptasensors hinges on achieving multiplexed detection for co-existing mycotoxins, enhancing sensor robustness for complex real-world matrices, and accelerating commercialization through cost-effective, user-friendly designs (101). The strategic integration of advanced nanomaterials and portable platforms will be pivotal in transitioning these sensitive biosensors from laboratory prototypes to reliable, field-deployable tools for on-site food safety monitoring.

### 4.2 ECL aptasensor

In recent years, significant advancements have been made in the development of ECL sensors for the detection of contaminants such as ZEN in various matrices. In 2019, a self-enhanced ECL sensor was fabricated by electrostatically assembling amine-functionalized Ru(bpy)_3_^2+^-doped SiO_2_ nanoparticles with nitrogen-doped graphene quantum dots. This innovative platform demonstrated the capability to detect ZEN in corn flour within a wide linear range of 10 fg/mL to 10 ng/mL, showcasing its potential for sensitive applications in food safety (102).

Building upon the foundation of luminescent materials, Luo et al. investigated self-enhanced ECL composite materials composed of nitrogen-doped graphene quantum dots and silica nanoparticles, specifically focusing on their interaction with Ru(bpy)_3_^2+^ (103). Their pioneering research revealed a remarkable low detection limit of 8.5 × 10^−16^ g/mL, further highlighting the efficacy of co-reactants and luminescent groups in enhancing the performance of ECL systems.

Continuing this trend of innovation, Xiang et al. reported in 2023 an ultra-low potential ECL sensor that operated based on a resonance energy transfer (RET) system (104). This advanced sensor achieved an unprecedented quantification range of 0.5 μg/mL to 0.1 fg/mL for ZEN, with an LOD at an astonishing 0.085 fg/mL, demonstrating its exceptional sensitivity and applicability in detecting trace amounts of contaminants (Figure 7D).

Together, these studies illustrate the significant strides made in the field of ECL sensors, combining novel materials and innovative methodologies to enhance detection limits and broaden quantification ranges, thereby contributing to advancements in safety monitoring in the food industry.

### 4.3 PEC aptasensor

Furthermore, Luo et al. (105) constructed a novel photoelectrochemical (PEC) aptasensor based on an *in situ*-formed composite of zinc oxide and nitrogen-doped graphene quantum dots. The sensor exhibited a wide linear range from 1.0 × 10^−4^ to 100 ng/mL, with an LOD of 3.3 × 10^−5^ ng/mL. Recovery results were consistent with those obtained by HPLC-MS. Another type of PEC aptasensor utilizes the synergistic effect of the Z-shaped BiOI/g-C_3_N_4_/Au heterojunction and Bi_2_S_3_ as a signal enhancer (106). It exhibits excellent sensitivity and selectivity for ZEN within a concentration range of 0.1 fg/mL to 1.0 ng/mL, with a detection limit of 0.087 fg/mL. The following year, a novel amplified PEC aptasensor was developed based on photoelectric activity and a donor-acceptor (D-A) conjugated covalent organic framework (COF) (107). By combining a target modulation-based competitive binding method with a multivariate signal amplification strategy, the sensor demonstrated excellent practical applicability, with a range from 0.1 pg./mL to 20 ng/mL, and an LOD of 24 fg/mL. In the same year, a signal photoelectrochemical aptasensor was developed that utilized cation exchange reaction amplification within the COF-367 NSs/CdS QD/Ag_2_S heterojunction. This approach, which did not require any biological assembly, addressed the issues of low signal intensity and false positives (108).

Cadmium sulfide (CdS) possesses a narrow band gap and a sufficiently negative conduction band potential, making it a promising photoactive material responsive to visible light. A PEC aptasensor constructed using a three-dimensional CdS/ethylenediamine nanowire network (3D CdS/EDA NWN) demonstrated high stability and practicality, achieving an LOD of 0.024 pg./mL (109). Similarly, sensors incorporating Cd-based materials utilized a Z-type NiTiO_3_/CdIn_2_S_4_ heterojunction as the photoactive layer, combined with a sandwich-structured PEC sensor featuring NiFe_2_O_4_ nanoparticles modified with ZEN-specific aptamers serving as the recognition probe (110). The magnetic-assisted separation strategy simplifies the experimental procedure, reduces false positives and false negatives, and achieved an LOD as low as 2.6 fg/mL. Wang et al. also developed a sandwich-type PEC aptasensor based on Cu_2_MoS_4_/CdS/In_2_S_3_ nanoclusters, which exhibited excellent stability, anti-interference capability, and an LOD as low as 2.38 fg/mL (111).

In summary, electrochemical aptasensors commonly immobilize aptamers onto conductive substrates, such as gold or carbon-based electrodes, through physical adsorption, thiol covalent bonding, or streptavidin–biotin affinity. The resulting recognition event is effectively transduced into an electrochemical signal. A variety of novel nanomaterials and detection strategies have been thoughtfully integrated into these sensors. Looking forward, the integration of electrochemical aptasensors with smart electronic systems represents a promising approach for achieving intelligent and automated mycotoxin monitoring.

## 5 Application of other technical applications in ZEN detection

Beyond conventional optical and electrochemical methods, several emerging techniques have shown considerable potential for ZEN detection. For example, a label-free chemiluminescence aptasensor was developed using molecular docking analysis to optimize multiple parameters, including aptamer truncation. The optimized sensor achieved an LOD of 2.85 ng/mL within a linear range of 1–100 ng/mL, representing a 6.7-fold improvement in sensitivity compared to the pre-optimized system (112).

A nanopore-based, label-free CRISPR/Cas12a system achieved effective signal amplification, with a low LOD of 6.52 fM (113). Another label-free approach utilized AuNPs as a cost-effective and rapid detection platform. After 8 rounds of SELEX screening, the assay was applied for on-site detection in animal feed, including dairy cow concentrate, pregnant sow feed, and laying hen feed, achieving an LOD of 12.5 nM (114).

MNPs have also been employed as separation and capture probes. In one study, MNPs served as capture probes in conjunction with TRFL nanoparticle-labeled complementary DNA as a signal probe, enabling the detection of ZEN over a concentration range of 0.001–10 ng/mL with an LOD of 0.21 pg./mL (26). In the same year, a lateral flow aptasensor was reported for ZEN detection within the range of 5–200 ng/mL, achieving an LOD of 20 ng/mL in corn samples (115). A related approach combined a high-loading aptamer-modified monolithic column with HPLC. This method employed a hydrophilic polyhedral oligomeric silsesquioxane (POSS)-based monolith prepared via photopolymerization and thiol bonding, providing high aptamer capacity, efficient preparation, and excellent specificity (116). The system achieved an LOD of 0.02 ng/mL and demonstrated strong selectivity, stability, and reproducibility, even in the presence of other mycotoxins.

It is noteworthy that an evanescent wave optical fiber aptasensor was reported for the first time, offering exceptional sensitivity, specificity, low cost, and compatibility with automation (117). This sensor consisted of a pixelated sensing layer and a microextraction layer and detected ZEN in corn flour extract across a range of 10 pM–10 nM, with an LOD of 18.4 ± 4.0 pM, more than 1,000 times lower than the regulatory limits established by China and the European Union. The sensor exhibited negligible cross-reactivity with other common mycotoxins and could be regenerated up to 28 times, significantly reducing operational costs. With a total measurement time of approximately 1 h, this system has shown strong potential for practical applications. Also notable is a chitosan nanofiber-based solid-phase extraction method developed using a modified freeze-drying process. This approach demonstrated good stability and repeatability, with a linear range of 0.06–10.0 μg/L and an LOD of 18.0 ng/L, enabling trace detection of ZEN in corn, wheat, and beer (118). Furthermore, a recently developed adaptive sensor, based on a glucose meter and driven by CRISPR/Cas12a, has achieved an LOD of 0.218 ng/mL and a detection range of 0.218–109.89 ng/mL (119). This innovation offers a novel approach to improving detection performance and enhancing accessibility for the public.

Several multiplexed detection platforms have been developed. Among them, Yang’s team designed a dual-focus aptasensor using microscale thermophoresis, in which ZEN and OTA aptamers were linked by a connector to form a bifunctional aptamer (120). This system simultaneously detected ZEN and OTA in corn oil, achieving an LOD of 0.12 nM for each, which is lower than that of many existing methods. Similarly, Chi et al. (121) developed mixed-mode aptamer-functionalized monolithic columns (Apt-MCs) based on hydrophobic and ionic interactions, enabling online specific recognition of ZEN, OTA, and AFB1 with LODs of 0.05, 0.03, and 0.05 μg/L, respectively. Additionally, a portable paper microfluidic aptasensor reported in 2023 allowed simultaneous multi-toxin detection using smartphone-based fluorescence capture and analysis (122). This system is simple, low-cost, and suitable for on-site use. It achieved an LOD of 0.44 ng/mL for ZEN with a linear range of 0.5–100 ng/mL and 0.098 ng/mL for OTA with a linear range of 0.1–50 ng/mL. Future iterations of this platform may incorporate enhanced portability and expanded multiplexing capabilities, highlighting its significant market potential.

These emerging detection methods utilize the high affinity, selectivity, and stability of aptamers, while incorporating advanced nanomaterials to enhance sensitivity, portability, and commercial viability (30, 123). In the future, integrating multiple detection principles could further improve performance and accelerate the translation of aptasensors from the laboratory to real-world field applications.

## 6 Conclusions and outlook

This review has examined the application of aptasensors for ZEN detection, addressing current challenges and proposing future research directions. Various types of aptasensors, including optical, electrochemical, and other emerging platforms, each present distinct advantages and limitations. It should be noted that the performance of these sensors fundamentally relies on the recognition elements at their core. Currently, several aptamers have been successfully developed and applied for ZEN detection. Based on current methodologies, future research should focus on the following aspects:

- Multiplexed detection capability. The simultaneous presence of multiple mycotoxins in real samples presents a common challenge. Most current aptasensors are designed for single-target detection, partly due to limitations in the selectivity and specificity of available aptamers. Developing reliable multiplexed aptasensors capable of detecting ZEN and other mycotoxins simultaneously will require dedicated research and specialized investment in aptamer screening and sensor integration.
- Improved sensor stability and robustness. The performance of aptasensors can be compromised by complex sample matrices and environmental variability. Improving stability requires the rational design of sensing interfaces, the incorporation of antifouling materials, and the implementation of robust signal amplification strategies. Developing dual-signal or self-calibrating detection modes presents a promising approach to increase reliability under real-world conditions.
- Accelerated commercialization and practical deployment. Most aptasensor research remains at the laboratory stage. Bridging the gap between research prototypes and commercially viable products will require cost-effective fabrication, simplified operation, and validation across diverse agricultural and food products. Improving sensitivity and throughput while maintaining affordability is essential to enable widespread on-site deployment.
- Applications of nanomaterials. Well-designed nanomaterials, such as metal nanoparticles, graphene derivatives, and MOFs, can significantly enhance sensor performance. These materials offer opportunities for signal amplification, improved bioreceptor immobilization, and increased selectivity, providing new pathways to overcome current detection limitations.
- Enhanced portability and point-of-need deployment. The ultimate value of aptasensors lies in their potential for rapid, on-site screening outside laboratory settings. Future research should prioritize the development of truly portable, user-friendly, and integrated detection platforms. This involves the miniaturization of readout systems (e.g., smartphone-based detectors, handheld potentiostats), the design of disposable or reusable test strips/cartridges, and the simplification of sample pretreatment steps. Leveraging advances in flexible electronics, microfluidics, and wireless communication can lead to the creation of compact, battery-operated devices capable of wireless data transmission. Such “lab-in-a-box” or even “lab-on-a-phone” systems would empower inspectors, farmers, and consumers to perform reliable ZEN detection directly at storage facilities, fields, markets, or homes, transforming food safety monitoring from a centralized lab-based activity into a distributed, real-time surveillance network.

In summary, aptasensors represent a dynamic and promising field of research for ZEN detection. Although antibody-based ELISA remains the gold standard for rapid screening at present, aptamer sensors have unique potential in terms of stability, cost, modifiable flexibility, and inter-batch consistency, and are an important direction for promoting the commercialization of the next-generation detection technologies. With ongoing innovations in materials, bioreceptor engineering, and device integration, these sensors are well-positioned to become powerful tools for food safety monitoring. We believe this review will serve as a valuable reference to support further advancements in sensor technology and contribute to the development of reliable, field-deployable detection systems.
